# Religion and suicide in Hungary: uncovering regional patterns and the protective effect of Catholicism (2000–2022)

**DOI:** 10.1186/s12889-026-27458-2

**Published:** 2026-04-30

**Authors:** Tamás Lantos, Zsanett Gabriella Urfi, Tibor András Nyári

**Affiliations:** Department of Medical Physics and Informatics, Faculty of Medicine, University of Szeged, 9 Korányi Alley, Szeged, 6720 Hungary

**Keywords:** Suicide mortality, Catholic religion, Religious affiliation, Protective factor, Cultural factors, Mental health, Hungary, Social cohesion, Regional variation, COVID-19

## Abstract

**Background:**

Suicide remains a critical public health issue in Hungary, a country with historically high suicide rates. This study investigates the relationship between regional religious affiliation and suicide mortality from 2000 to 2022, shedding light on the influence of social and cultural factors in suicide prevention.

**Methods:**

Data were sourced from the national population register and the three most recent censuses. We applied quantile and gamma regressions to explore the associations between age-standardised suicide rates and the age-standardised incidence rates of religious affiliation across regions, reflecting the religious composition.

**Results:**

Our findings confirm a continued nationwide decline in suicide mortality, yet regional variations persist, particularly in relation to religious affiliation. Suicide rates were significantly lower in regions with higher proportions of Roman Catholics (*p* = 0.008) and higher in areas with a greater percentage of non-religious individuals (*p* = 0.007). These trends held steady before and during the COVID-19 pandemic. Notably, the geographic distribution of Catholics and suicide rates displayed opposing patterns across Hungary’s eight statistical regions.

**Conclusions:**

These results, based on robust census data, suggest that Catholic affiliation may have a protective effect against suicide, potentially through enhanced social cohesion and community support. Understanding these regional patterns can inform more targeted mental health interventions and highlight the critical role of social and cultural factors in suicide prevention.

## Introduction 

The Hungarian suicide mortality rate had consistently been among the highest in Europe for decades. Although suicide deaths nearly halved in Hungary during 2000–2019, they remained the leading external cause of death, with more than 2,600 deaths per year on average [1]. Nevertheless, the distribution of suicide mortality in Hungary has always been characterised by regional inequalities.

Regional differences in suicide rates in Hungary were previously reported by Rihmer and colleagues [2]. Another Hungarian research group found an elevated suicide risk among both men and women living on the Great Plain (in eastern and south-eastern Hungary) and concluded that this effect could not be explained by education, marital status, or other demographic factors [3].

Protective factors against suicide have been described, including effective mental health care, strong personal relationships, supportive social networks, life skills and adaptability, the practice of positive coping strategies, and religious or spiritual beliefs [4].

During the 1990 s, religious affiliation increased in Hungary, and its protective role was demonstrated in a Hungarian study [5].

The most accurate data on the distribution of religious affiliation are obtained through a census. However, data on religious affiliation were collected only once in Hungary in the mid-20th century, in 1949. After the 1949 census, religious affiliation was recorded only in the new millennium, in 2001, 2011, and 2022. A census is conducted every 10 years in Hungary; however, the most recent one was postponed by a year due to the coronavirus pandemic.

In Hungary, the COVID-19 pandemic led to an increase in excess deaths [6]. Nevertheless, the COVID-19 pandemic has influenced the regional pattern of suicide in Hungary, as significant increases in suicide rates were observed in the two regions with the lowest COVID-19 mortality rates [7].

In Hungary, previous research has highlighted the importance of social, cultural, and health-related factors in explaining regional differences in suicide rates. Kopp et al. [8] demonstrated that religiosity and the integrative role of religious communities are significantly associated with mental health indicators, contributing to differences in morbidity between traditionally Catholic and Protestant regions.

From a sociological perspective, religion can also be understood as a contextual characteristic of communities. Following Durkheim’s framework, higher levels of religious affiliation within a region may reflect stronger social integration and moral regulation, which can exert a protective effect against suicide.

Several Hungarian studies have emphasised that regional variation in suicide rates is shaped by multiple interacting factors: Moksony and Hegedűs [9] highlighted the role of cultural background, Zonda et al. [5] emphasised demographic and social structure, while Rihmer et al. [2] pointed to regional differences in the diagnosis and treatment of psychiatric disorders.

In this context, regional religious composition may serve as an additional indicator of broader social and cultural environments that influence suicide risk. In our study, we investigated the pattern of religious affiliation and suicide risk in Hungary using data from vital registers and censuses over the 23-year period from 1 January 2000 to 31 December 2022.

## Methods

### Study population and suicide data

The study period spanned 1 January 2000 to 31 December 2022 and was divided into three sub-periods: the first decade (2000–2009), the second decade (2010–2019), and the pandemic period (2020–2022). Data from the 2001, 2011, and 2022 censuses were used to calculate person-years for the two pre-pandemic decades and the pandemic period, respectively. Similarly, the census database included data on religious affiliation, broken down by region and 10-year age groups.

The analyses included the populations of historical churches (the Roman Catholic Church, the Reformed Church of Hungary, and the Evangelical-Lutheran Church), the non-religious population (“Not belonging to any church”), and non-respondents, respectively. Other religions (e.g., Orthodox, Baptist) were grouped as the ‘other religious’ population. The following abbreviations are used in the text: Catholics for individuals affiliated with the Roman Catholic Church, Calvinists for those affiliated with the Reformed Church of Hungary, and Lutherans for those affiliated with the Evangelical-Lutheran Church.

Data on suicide deaths were obtained from the nationwide population register published by the Hungarian Central Statistical Office [10]. Suicide mortality is recorded in Hungary’s vital register, which ranks among the highest in the world in terms of statistical performance.

These data were classified according to the International Classification of Diseases, 10th Revision (ICD-10), with codes for ‘intentional self-harm’ corresponding to X60–X84 and Y87.0.

Both population and suicide deaths were stratified by region and 10-year age groups for all three censuses. Annual suicide deaths were then aggregated according to the defined sub-periods.

Suicide deaths were aggregated within three predefined periods (2000–2009, 2010–2019, and 2020–2022). For each period, population denominators were derived from the corresponding census (2001, 2011, and 2022, respectively), stratified by age group and region. These census populations were used to approximate person-years at risk for each period, under the assumption of a relatively stable population structure within each interval.

Although population size and composition may vary within periods, age-standardisation using the Revised European Standard Population mitigates the impact of such changes, allowing for meaningful comparison across regions and time.

Suicide rates (SRs) were expressed as per 100,000 person-years. The regional suicide rates were directly standardised by age [11] using the Revised European Standard Population (RESP) published in 2013 [12]. This standardisation facilitated comparison of rates over time by removing the effect of age composition, yielding age-standardised suicide rates (ASSRs).

Similarly, incidence rates (IRs) of religious affiliation were directly standardised by age for each region. The resulting age-standardised incidence rate (ASIR) represents the summary rate that would have been observed, given the schedule of age-specific rates, in a population with the age composition of a reference population.

**Fig. 1:**
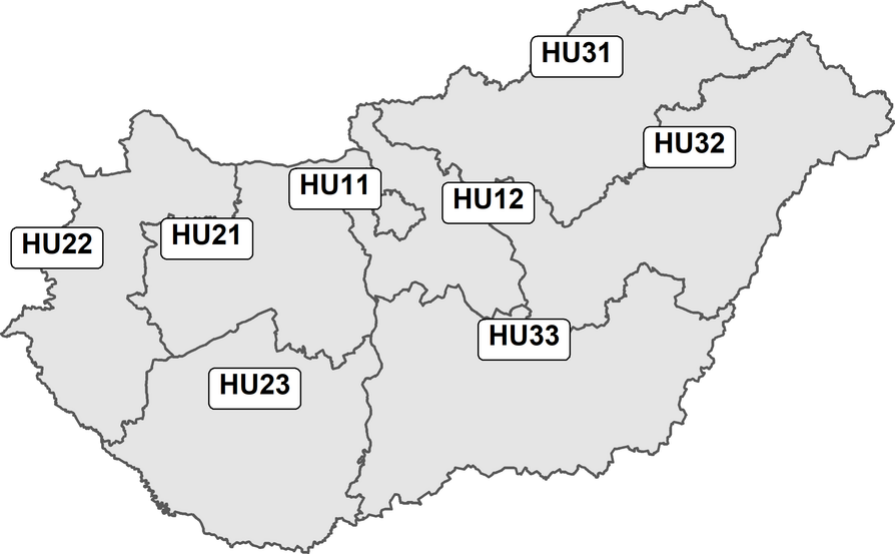
The Hungarian regional administration using the second level of the NUTS 2021 classification: Budapest (HU11), Pest County (HU12), Central Transdanubia (HU21), Western Transdanubia (HU22), Southern Transdanubia (HU23), Northern Hungary (HU31), Northern Great Plain (HU32) and Southern Great Plain (HU33)

Territorial units were defined using the second level of the NUTS 2021 classification (Nomenclature of Territorial Units for Statistics; 2021 revision) [13]. Hungary was divided into eight regions (Fig. 1): Budapest (HU11), Pest County (HU12), Central Transdanubia (HU21), Western Transdanubia (HU22), Southern Transdanubia (HU23), Northern Hungary (HU31), the Northern Great Plain (HU32), and the Southern Great Plain (HU33).

The relationship between age-standardised suicide rates (ASSRs) and age-standardised incidence rates (ASIRs) of religious affiliation was examined using quantile regression [14] and Gamma regression models [15]. To examine the association between suicide mortality and religious affiliation, both quantile regression and Gamma regression models were applied. The median (50th percentile) was used as the default quantile in the analyses. The regression coefficients (β’s) and their 95% confidence intervals (95% CIs) were calculated. Nevertheless, sensitivity analyses were also conducted using the lower (25%) and upper (75%) quantiles. Since the suicide rate distribution is skewed, we employed Gamma regression to ensure robust estimation, which handles the heteroscedasticity effectively. In the multivariable models, the ASIRs of Catholics, Calvinists, Lutherans, non-religious individuals, and non-respondents were included as explanatory variables, with additional adjustment for time period and region.

The analyses were conducted using region-period level data, where each region contributed one observation per study period (2000–2009, 2010–2019, and 2020–2022). Temporal variation was therefore incorporated through the inclusion of multiple periods, rather than modelling time as a continuous variable.

To assess the consistency of associations across time, additional analyses were performed separately for each period.

*P*-values of less than 0.05 were considered statistically significant. All statistical analyses were conducted using STATA 17.0 (StataCorp LP, College Station, TX, USA).

## Results

Between 2000 and 2022, 51,775 suicides occurred in Hungary, corresponding to an annual rate of 22.7 per 100,000 person-years (95% CI: 22.5–22.9). However, 493 suicides lacked a registered residence and were excluded from the analyses. During 2000–2009, 2010–2019, and 2020–2022, 27,104, 19,757, and 4,914 suicides were recorded in Hungary, corresponding to annual rates of 26.8, 20.0, and 16.9 per 100,000 person-years, respectively. Crude suicide rates (SRs) decreased significantly over the 23 years of study. Age-standardised suicide rates (ASSRs) decreased significantly from 28.8 (95% CI: 27.8–29.9) to 16.5 per 100,000 person-years (95% CI: 15.2–17.9). Across all three periods, suicide rates were highest in the 40–59 and 60–79-year age groups, although the trends in these groups moved in opposite directions. Suicide rates declined in the 40–59-year age group but increased in the 60–79-year age group (Table 1).

**Table 1:** The number of suicide deaths and religious demographic characteristics by age of the Hungarian population during 2000–2022

Census/Age group	Population	Suicide	%	Catholics	%	Calvinists	%	Lutherans	%	Non-religious people	%	Non-respondents	%
2001 (years considered for suicides: 2000–2009)													
Under 20 years	2 363 545	485	1.8	1 140 664	48.3	324 236	13.7	54 618	2.3	525 317	22.2	293 690	12.4
20 to 39 years	2 905 884	5 177	19.3	1 472 474	50.7	406 562	14.0	70 953	2.4	544 422	18.7	371 305	12.8
40 to 59 years	2 847 327	11 887	44.3	1 622 895	57.0	476 482	16.7	88 888	3.1	323 473	11.4	304 541	10.7
60 to 79 years	1 801 776	7 014	26.1	1 142 869	63.4	360 847	20.0	76 327	4.2	81 581	4.5	117 452	6.5
80 years and over	279 783	2 268	8.5	180 059	64.4	54 669	19.5	13 919	5.0	8 576	3.1	17 345	6.2
Hungary	10 198 315	26 831	100	5 558 961	54.5	1 622 796	15.9	304 705	3.0	1 483 369	14.5	1 104 333	10.8
2011 (years considered for suicides: 2010–2019)													
Under 20 years	2 041 193	317	1.6	668 432	32.7	207 204	10.2	35 224	1.7	478 272	23.4	610 753	29.9
20 to 39 years	2 810 449	3 333	17.0	941 062	33.5	273 998	9.7	48 779	1.7	632 086	22.5	853 577	30.4
40 to 59 years	2 754 875	7 868	40.2	1 092 101	39.6	314 550	11.4	56 015	2.0	478 038	17.4	760 912	27.6
60 to 79 years	1 931 879	6 150	31.4	954 946	49.4	291 618	15.1	59 559	3.1	193 179	10.0	403 028	20.9
80 years and over	399 232	1 913	9.8	215 381	53.9	66 084	16.6	15 516	3.9	24 834	6.2	70 574	17.7
Hungary	9 937 628	19 581	100	3 871 922	39.0	1 153 454	11.6	215 093	2.2	1 806 409	18.2	2 698 844	27.2
2022 (years considered for suicides: 2020–2022)													
Under 20 years	1 879 284	77	1.6	452 963	24.1	161 794	8.6	30 612	1.6	415 715	22.1	784 717	41.8
20 to 39 years	2 315 049	742	15.2	535 133	23.1	185 708	8.0	31 057	1.3	451 439	19.5	1 057 020	45.7
40 to 59 years	2 864 605	1 685	34.6	838 629	29.3	267 104	9.3	48 435	1.7	445 841	15.6	1 200 014	41.9
60 to 79 years	2 110 505	1 792	36.8	852 602	40.4	262 901	12.5	51 606	2.4	213 441	10.1	694 963	32.9
80 years and over	434 191	574	11.8	207 292	47.7	66 475	15.3	14 793	3.4	23 174	5.3	115 819	26.7
Hungary	9 603 634	4 870	100	2 886 619	30.1	943 982	9.8	176 503	1.8	1 549 610	16.1	3 852 533	40.1
Overall	29 739 577	51 282		12 317 502	41.4	3 720 232	12.5	696 301	2.3	4 839 388	16.3	7 655 710	25.7

**Fig. 2:**
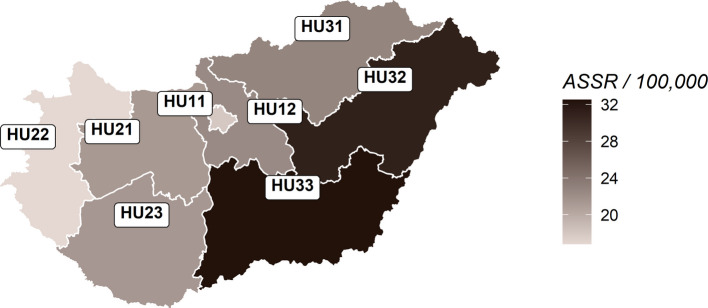
NUTS2 regions of Hungary coloured by age-standardised suicide rates (ASSRs) per 100,000 population) during 2000–2022

There was a statistically significant regional variation in ASSRs (β = 1.6, 95% CI: 0.02 to 3.13; *p* = 0.048), where the regression coefficient represents the difference in age-standardised suicide mortality rates (per 100,000 person-years) associated with regional categories in the model. The lowest ASSRs were observed in the Western Transdanubia region, while the highest were in the Southern Great Plain region (Fig. 2). The Northern Great Plain region also showed high ASSRs, with the highest rates recorded during the pre-pandemic decades (2000–2019).

### Census data

Hungary’s population declined from 10,198,315 in 2001 to 9,937,628 in 2011 and 9,603,634 in 2022. Table 1 summarises suicide deaths and religious affiliation by age group. Table 2 summarises national and regional data on religious denominations from the census.

**Table 2:** The number of suicide deaths and religious demographic characteristics by regions of the Hungarian population during 2000–2022. Codes: *HU11* Budapest, *HU12* Pest County, *HU21* Central Transdanubia, *HU22* Western Transdanubia, *HU23* Southern Transdanubia, *HU31* Northern Hungary, *HU32* Northern Great Plain, *HU33* Southern Great Plain

Census/Region	Population	Suicide	Catholics	%	Calvinists	%	Lutherans	%	Non-religious population	%	Non-respondents	%	Other religions	%
2001 (years considered for suicides: 2000–2009)														
HU11	1 777 921	3 601	837 548	47.1	224 169	12.6	46 449	2.6	347 209	19.5	286 584	16.1	35 962	2.0
HU12	1 083 877	2 633	587 486	54.2	173 602	16	41 346	3.8	129 983	12	135 837	12.5	15 623	1.4
HU21	1 119 480	2 592	632 802	56.5	152 157	13.6	34 998	3.1	173 660	15.5	117 267	10.5	8 596	0.8
HU22	1 004 300	1 901	773 506	77	35 271	3.5	53 317	5.3	56 438	5.6	81 114	8.1	4 654	0.5
HU23	993 522	2 454	662 495	66.7	92 323	9.3	23 485	2.4	114 618	11.5	91 902	9.3	8 699	0.9
HU31	1 290 392	3 284	799 891	62	220 358	17.1	18 481	1.4	128 920	10	110 601	8.6	12 141	0.9
HU32	1 551 171	5 276	555 575	35.8	547 062	35.3	19 240	1.2	275 272	17.7	137 979	8.9	16 043	1.0
HU33	1 377 652	5 090	709 658	51.5	177 854	12.9	67 389	4.9	257 269	18.7	143 049	10.4	22 433	1.6
Hungary	10 198 315	26 831	5 558 961	54.5	1 622 796	15.9	304 705	3.0	1 483 369	14.5	1 104 333	10.8	124 151	1.2
2011 (years considered for suicides: 2010–2019)														
HU11	1 729 040	2 776	517 762	29.9	146 756	8.5	30 293	1.8	395 964	22.9	585 475	33.9	52 790	3.1
HU12	1 217 476	2 164	445 106	36.6	134 848	11.1	32 564	2.7	220 299	18.1	355 431	29.2	29 228	2.4
HU21	1 082 313	1 927	431 198	39.8	107 071	9.9	24 450	2.3	201 864	18.7	303 322	28	14 408	1.3
HU22	986 793	1 464	572 380	58	28 866	2.9	40 253	4.1	86 769	8.8	249 712	25.3	8 813	0.9
HU23	934 109	1 727	459 322	49.2	64 574	6.9	17 219	1.8	145 263	15.6	234 947	25.2	12 784	1.4
HU31	1 197 575	2 381	557 558	46.6	154 573	12.9	12 425	1	161 802	13.5	292 151	24.4	19 066	1.6
HU32	1 492 587	3 622	392 512	26.3	395 178	26.5	13 243	0.9	312 956	21	352 438	23.6	26 260	1.8
HU33	1 297 735	3 520	496 084	38.2	121 588	9.4	44 646	3.4	281 492	21.7	325 368	25.1	28 557	2.2
Hungary	9 937 628	19 581	3 871 922	39.0	1 153 454	11.6	215 093	2.2	1 806 409	18.2	2 698 844	27.2	191 906	1.9
2022 (years considered for suicides: 2020–2022)														
HU11	1 685 342	757	408 823	24.3	126 920	7.5	26 162	1.6	324 157	19.2	749 282	44.5	49 998	3.0
HU12	1 333 533	602	378 260	28.4	127 504	9.6	29 490	2.2	215 027	16.1	552 558	41.4	30 694	2.3
HU21	1 055 648	519	311 825	29.5	88 926	8.4	19 593	1.9	181 362	17.2	437 817	41.5	16 125	1.5
HU22	976 258	382	441 540	45.2	29 501	3	34 987	3.6	86 147	8.8	371 246	38	12 837	1.3
HU23	855 423	388	321 308	37.6	49 727	5.8	13 316	1.6	124 936	14.6	332 634	38.9	13 502	1.6
HU31	1 091 375	547	396 575	36.3	118 789	10.9	9 701	0.9	130 695	12	416 830	38.2	18 785	1.7
HU32	1 404 331	811	287 644	20.5	312 551	22.3	10 498	0.7	255 260	18.2	512 466	36.5	25 912	1.8
HU33	1 201 724	864	340 644	28.3	90 064	7.5	32 756	2.7	232 026	19.3	479 700	39.9	26 534	2.2
Hungary	9 603 634	4 870	2 886 619	30.1	943 982	9.8	176 503	1.8	1 549 610	16.1	3 852 533	40.1	194 387	2.0

Table 2 shows that affiliation with historical churches declined significantly, whereas the proportions of non-religious individuals and non-respondents increased, based on census data. Between 2001 and 2022, Hungary’s population declined by 595,000. In all three censuses, Catholics comprised the largest proportion of respondents, followed by Calvinists. During this period, Catholic respondents declined by over 2.6 million, Calvinists by more than 678,000, and Lutherans by 128,000. Between 2001 and 2022, the proportion of Catholics fell from 54.5% to 30.1%. The proportions of Calvinists and Lutherans also declined, based on census data. Calvinists declined from 15.9% to 9.8%, and Lutherans from 3.0% to 1.8%.

Table 2 shows that, as the Christian population declined, the proportions of non-religious individuals and non-respondents increased significantly. Across all three censuses, about 1.5 million Hungarians were unaffiliated with any religious community or denomination. The proportion of non-religious individuals ranged from 14.5% to 18.2%. The proportion of non-respondents to the religious denomination question increased from 10.8% in 2001 to 40.1% in 2022. Among all age groups under 60 years, non-respondents accounted for more than 40%.

**Fig. 3:**
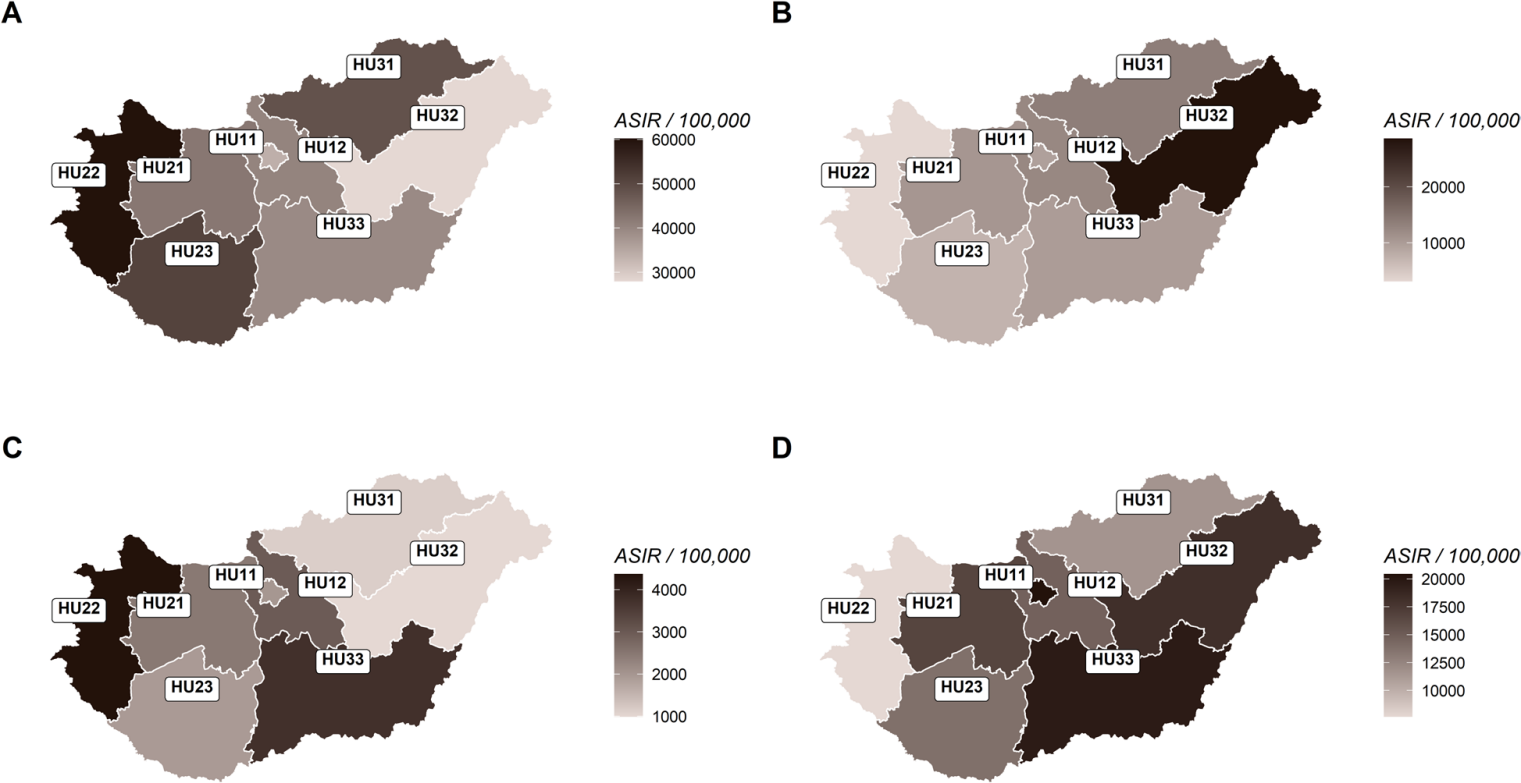
The regional age-standardised incidence rates (ASIRs) of: **A** Catholics, **B** Calvinists, **C** Lutherans, **D** Non-religious people

Between the three censuses, the proportion of Christians in all eight regions fell by almost half. Western and Southern Transdanubia showed a slightly smaller decline in the proportion of Catholics. The proportion of Catholics in the Budapest agglomeration decreased non-significantly, whereas in Eastern Hungary it declined significantly. At all three censuses, the highest and lowest age-standardised incidence rates (ASIRs) of the Catholic religion were observed in Western Transdanubia (HU22) and the Northern Great Plain (HU32), respectively. Regional ASIRs of religious affiliation in Hungary are presented in Fig. 3A–D.

Figures 2 and 3A show that age-standardised suicide rates (ASSRs) and age-standardised incidence rates (ASIRs) of Catholics varied in opposite directions across the eight Hungarian regions. Western Transdanubia (HU22) had the highest Catholic ASIR and lowest ASSR, whereas the Northern Great Plain (HU32) showed the lowest Catholic ASIR and one of the highest ASSRs. In Budapest (HU11), both ASSR and Catholic ASIR were the second lowest, differing from the patterns observed in the other regions. The highest ASIRs of non-religious individuals were observed in the Northern Great Plain (HU32) and the Southern Great Plain (HU33) (Fig. 3D).

Quantile regression was applied to assess the relationship between regional ASSRs and ASIRs of religious affiliation, despite the lack of data on individual suicide cases’ religion. Regional ASSRs were significantly negatively associated with Catholic ASIRs (β = –0.00029, 95% CI: –0.00055 to –0.00033; *p* = 0.029) and positively associated with non-religious ASIRs (β = 0.0006, 95% CI: 0.0002 to 0.0010; *p* = 0.002).

Regional ASSRs showed a similar, but not statistically significant, association with Calvinist ASIRs (β = 0.00048, 95% CI: –0.00013 to 0.0011; *p* = 0.11) and non-respondent ASIRs (β = −0.00056, 95% CI: −0.00195 to 0.002085; *p* = 0.405) (Table 5).

These associations remained similar in the pre-pandemic and pandemic periods, whereas regional ASSRs showed no relationship with Lutheran ASIRs (β = 0.0015, 95% CI: –0.00084 to 0.0038; *p* = 0.19).

Similar relationships between regional ASSRs and ASIRs of religious affiliation were found using lower (0.25) and upper (0.75) quantiles in the quantile regression models. The negative association between regional ASSRs and Catholic ASIRs, as well as the positive association with non-religious ASIRs, remained statistically significant in the Gamma regression model. In the multivariable models including religious affiliation variables, only Catholic ASIRs remained significantly associated with ASSRs. All models demonstrated satisfactory goodness-of-fit statistics. Tables 3 and 4 summarise the regional age-standardised suicide rates (ASSRs) and age-standardised incidence rates (ASIRs) of religious affiliation by census year (2001, 2011, and 2022), as well as overall rates in Hungary. In addition, the regression coefficients from the quantile and Gamma regression models are summarised in Table 5.

**Table 3:** The regional age-standardised suicide rates (ASSRs) and age-standardised incidence rates (SIRs) of religious affiliation by censuses (2001, 2011 and 2022) in Hungary. Codes: *HU11* Budapest, *HU12* Pest County, *HU21* Central Transdanubia, *HU22* Western Transdanubia, *HU23* Southern Transdanubia, *HU31* Northern Hungary, *HU32* Northern Great Plain, *HU33* Southern Great Plain

Census/Region	ASSR	SE	Catholics	SE	Calvinists	SE	Lutherans	SE	Non-religious population	SE	Non-respondents	SE	Other religions	SE
2001 (years considered for suicides: 2000–2009)														
HU11	21.0	1.02	47439	163	12743	84	2657	39	19155	96	15972	92	2034	34
HU12	27.5	1.60	55168	231	16447	127	3952	63	11001	91	11982	102	1451	37
HU21	25.9	1.52	57691	234	13985	116	3275	57	14267	101	10015	92	767	27
HU22	20.1	1.35	77507	280	3566	60	5405	75	5264	65	7803	85	455	21
HU23	26.7	1.58	67359	264	9501	100	2436	51	10867	94	8958	92	879	30
HU31	27.4	1.39	62651	223	17296	117	1498	35	9351	76	8251	76	954	28
HU32	39.8	1.61	36643	157	36440	158	1308	30	16099	91	8447	71	1062	27
HU33	40.6	1.66	52161	195	13304	99	5127	62	17664	101	10082	82	1662	35
2011 (years considered for suicides: 2010–2019)														
HU11	16.1	0.88	30098	131	8545	70	1767	32	22726	109	33824	136	3041	41
HU12	19.3	1.22	37450	178	11366	98	2751	48	17403	112	28660	150	2371	43
HU21	18.3	1.23	40278	194	10034	97	2307	47	18348	121	27716	155	1318	34
HU22	14.7	1.10	58278	242	2944	54	4122	65	8694	87	25078	153	884	29
HU23	18.3	1.28	49237	228	6939	86	1859	45	15546	120	25050	158	1368	37
HU31	20.1	1.20	46815	197	12983	103	1054	30	13360	98	24201	137	1587	35
HU32	26.0	1.26	26749	135	26983	135	915	25	20381	109	23229	121	1744	33
HU33	27.3	1.33	38284	170	9417	84	3478	52	21634	120	24978	133	2210	41
2022 (years considered for suicides: 2020–2022)														
HU11	14.5	1.51	24136	118	7508	66	1553	30	19405	101	44454	157	2943	40
HU12	15.8	1.91	28699	147	9661	85	2238	41	15874	102	41233	171	2296	40
HU21	16.0	2.05	29213	164	8381	88	1845	42	17433	120	41603	192	1525	36
HU22	12.5	1.84	44799	211	3014	55	3579	60	9014	90	38286	191	1307	35
HU23	14.0	2.04	36704	203	5679	80	1521	42	15139	125	39373	206	1584	41
HU31	16.5	2.06	36139	179	10832	98	882	28	12055	98	38368	179	1724	38
HU32	19.3	1.96	20538	120	22348	125	752	23	18112	107	36405	155	1844	35
HU33	22.7	2.25	27781	149	7346	77	2666	46	19769	120	40222	176	2216	42

**Table 4:** The overall regional age-standardised suicide rates (ASSRs) and age-standardised incidence rates (SIRs) of religious affiliation during 2000–2022 in Hungary. Codes: *HU11* Budapest, *HU12* Pest County, *HU21* Central Transdanubia, *HU22* Western Transdanubia, *HU23* Southern Transdanubia, *HU31* Northern Hungary, *HU32* Northern Great Plain, *HU33* Southern Great Plain

Region	ASSR	SE	Catholics	SE	Calvinists	SE	Lutherans	SE	Non-religious population	SE	Non-respondents	SE	Other religions	SE
(years considered for suicides: 2000–2022)														
HU11	17.9	0.61	34,096	80	9619	42	2000	20	20,480	59	31,161	75	2644	22
HU12	21.8	0.88	39,512	105	12,228	59	2910	29	14,988	60	28,298	86	2063	23
HU21	21.0	0.87	42,631	114	10,812	58	2475	28	16,780	66	26,113	87	1189	19
HU22	16.6	0.78	60,446	142	3166	32	4370	38	7662	47	23,482	86	874	16
HU23	21.3	0.91	51,920	135	7450	51	1956	27	13,808	65	23,614	89	1253	21
HU31	22.9	0.84	49,292	117	13,878	62	1156	18	11,563	52	22,717	77	1394	19
HU32	30.8	0.91	28,197	80	28,684	81	994	15	18,324	59	22,276	69	1526	18
HU33	32.3	0.96	39,960	100	10,096	50	3773	31	19,796	66	24,371	76	2004	22

**Table 5:** Association between age-standardised suicide rates (ASSRs) and age-standardised incidence rates (ASIRs) of religious affiliation (quantile and Gamma regression). This table reports regression coefficients (β’s), 95% confidence intervals (95% CIs), and *p*-values for both quantile regression (median) and Gamma regression models. Significant *p*-values (*p* < 0.05) are indicated in bold

Variable/Religion	regression coefficients (β) of quantile regression	95% CI		*p*-value	regression coefficients (β) of gamma regression	95% CI		*p*-value
Catholic	−0.00029	−0.00055	−0.00003	**0.029**	−8.7 × 10^−^⁶	−1.6 × 10^–5^	−1.6 × 10^−^⁶	**0.015**
Calvinist	0.00049	−0.00013	0.00110	0.115	7.5 × 10⁻⁷	−2.4 × 10^−^⁶	1.7 × 10^–5^	0.138
Lutheran	0.00152	−0.00084	0.00389	0.194	1.7 × 10⁻^5^	−3.6 × 10^–5^	7.0 × 10^–5^	0.538
Non-religious	0.00055	0.00022	0.00088	**0.002**	1.5 × 10⁻^5^	1.3 × 10^−^⁶	3.0 × 10⁻^5^	**0.034**
Non-respondents	−0.00057	−0.00196	0.00083	0.405	−3.9 × 10⁻⁶	−3.8 × 10^–5^	3.8 × 10^–5^	0.819
Other religions	0.00273	−0.00595	0.01141	0.518	7.7 × 10⁻^5^	−1.2 × 10^–4^	2.7 × 10^–4^	0.440

## Discussion

### Main findings

This study confirmed a downward trend in suicide mortality in Hungary. A significant negative correlation was found between age-standardised suicide mortality and Catholic ASIRs, and a significant positive correlation with non-religious ASIRs. In the multivariable models, Catholic ASIRs remained the only significant protective factor. In other words, higher Catholic ASIRs were associated with lower regional ASSRs. Thus, Catholic religiosity could be a protective factor in the aetiology of suicide. These associations remained similar across the pre-pandemic and pandemic periods of COVID-19.

### Comparison with other studies

More than a century ago, Durkheim [16] argued that religion protects against suicide not through its specific dogmas, but through its function as a cohesive social institution. He emphasised that higher levels of social integration and moral regulation within religious communities create a collective protective effect for individuals.

However, empirical tests of this hypothesis have yielded inconsistent results, largely due to differences in the operationalisation of these concepts. Some studies have used denominational affiliation (e.g., comparing Catholics and Protestants) as a proxy for integration and regulation, while others have focused on indicators of religious participation, such as church attendance or the subjective importance of faith [17, 18].

These approaches capture different dimensions of religiosity, which may explain the apparent contradictions in the literature. While some studies find differences between denominations, others suggest that the I; ntensity of religious involvement is a stronger predictor of suicide risk than nominal affiliation [19–21].

In line with this tradition, Moksony [22] applied an ecological perspective, focusing on regional patterns of social integration rather than individual-level religiosity. Similarly, in the present study, we use regional religious affiliation as an ecological indicator that may reflect broader processes of social integration and moral regulation, rather than measuring these constructs directly.

In Europe and developed nations, there has been a growing disengagement from religion [23, 24]. The results of the last three censuses show a similar trend in Hungary [25].

The census questionnaire permitted respondents to answer the religious denomination question freely. In 2001, 10.8% of the population left the voluntary religion question unanswered, while this increased to 40.1% in 2022. After being removed in 1949, the question on religious affiliation was reinstated in the census questionnaire in 2001. Census data were collected solely through traditional paper-based questionnaires at that time. In 2011, the option to fill out the census online was introduced. While only 20% of the population completed the census questionnaire online in 2011, this proportion exceeded 70% by 2022 [25]. Alongside the decline of historical churches, the number and proportion of individuals identifying with other Christian denominations and religious communities have notably increased.

Zonda et al. demonstrated that a higher proportion of individuals affiliated with any denomination was associated with a reduced suicide risk, compared to those not belonging to a religious community in Hungarian communities [26]. However, our study identified a significant negative correlation between suicide and the incidence of Catholics, while significant positive correlations were found with the incidence of Calvinists and non-religious individuals in Hungary. Our findings are consistent with those of Moksony and Hegedűs, who found that the risk of suicide is higher among Calvinists than Catholics in Hungary [9]. Moksony and Hegedűs further observed that ‘deeper involvement in the church community decreases suicide risk for Catholics but increases it for Protestants’ [27].

VanderWeele et al. reported that attending religious services at least once per week was associated with a suicide rate approximately five times lower among Catholics compared to those who never attended [28]. In a previous study, Stack and Lester reported that higher church attendance was associated with a lower suicide rate [29]. Additionally, they found that the influence of religiosity on suicide ideation was independent of education, gender, marital status, and age. In contrast, O'Reilly and Rosato found in a longitudinal study of over 1 million individuals that the suicide risk was similar for those with and without religious affiliation, and that Catholics did not exhibit a lower risk of suicide [30].

During the coronavirus pandemic, due to government measures, the possibility of attending church services and other community gatherings was reduced, and many of these moved online.

Kanol and Michalowski did not find that the pandemic itself influenced religiosity in Germany [31]. However, the measure of religiosity they used is sensitive to increases in religious quest but does not capture more institutionalised religious practices, such as attendance at religious services. Similarly, Upenieks found that religious and spiritual struggles were somewhat common among Americans during COVID-19 [32]. However, both psychological distress and self-rated health were more favourable when religious and spiritual matters were discussed very frequently, several times a week or more.

The impact of the coronavirus pandemic varied across different regions of Hungary [33]. In some more economically developed regions with lower suicide rates, the number of suicides increased significantly during the pandemic. A recent study reported a significant relative increase in suicide mortality during the first year of the pandemic compared to pre-pandemic suicide rates in Budapest [7]. In our study, we found that age-standardised suicide rates remain low in the capital, but the age-standardised proportion of Catholics has halved from 2001 to 2022, which might partly explain the relative increase in suicide rates during the first year of the pandemic in Budapest.

In a recent Hungarian online study, Rixer and Ferenczi found that individuals who regularly practiced their religion in an institutional setting during the coronavirus pandemic reported significantly better mental and social well-being compared to those who were not regular religious practitioners [34]. On the contrary, in our study, the proportion of the population belonging to historical Christian churches has significantly decreased, while the proportion of the non-religious population has significantly increased over the past 23 years in Hungary. These trends were not influenced by the COVID-19 pandemic. Nevertheless, the Catholic religion remained a protective factor in the aetiology of suicide during the pandemic.

The incidence of religious affiliation also shows regional variations in Hungary, according to the censuses (Table 3). Moksony reported that the territorial distribution of suicides in Hungary exhibits a peculiar temporal constancy [35]. This form of deviant behaviour has been quite common in the Southern Great Plain region for many decades, while it has traditionally been relatively rare in the western regions. Some researchers explain this phenomenon by cultural differences across various parts of the country—differences in values and norms related to suicide between regions.

Additionally, Béla Hamvas described the Hungarian national character in his book *Five Geniuses* [36]. The five Hungarian national characters reflect the complex structure of the seven European substructures. The Hungarian sub-characters are as follows: the ‘genius’ of the South, the West, the North, the East, and Transylvania.

The ‘genius of the West’ reflects Western European civilisation and characterises Western Hungary, while the ‘genius of the East’ corresponds to the Hungarian Great Plain (both Northern and Southern), which embodies the ancient nomadic mentality.

Nevertheless, Zonda mentioned in his book that the Hungarians have never been deeply religious, believing people, at least not in the way that the Poles, Spaniards, Portuguese, or South Americans are Catholic [37]. The assumption that there may be a correlation between a high suicide rate and lukewarm religiosity is reasonable. Religion is an essential element of culture. In the Great Plains, the proportion of people following the Reformed religion is much higher than in the rest of the country. He also mentioned that the divorce rate is much higher in the Great Plains, which may be linked to the higher suicide rate in the region. In addition, Catholic-dominant regions, particularly Western and Central Transdanubia, tend to exhibit more favourable socioeconomic indicators compared to the national average. According to HCSO data [38], these regions are characterised by higher GDP per capita, stronger industrialisation, and higher employment levels. In contrast, regions of the Great Plain and Northern Hungary – where Catholic affiliation is lower – have historically shown less favourable socioeconomic conditions, including higher unemployment and lower average income [38].

These regional disparities suggest that the observed association between Catholic affiliation and lower suicide rates may partly reflect underlying socioeconomic differences. However, previous Hungarian studies have reported elevated suicide risk in the Great Plain even after adjusting for demographic factors, indicating that such differences may not be fully explained by socioeconomic composition alone. Therefore, religious affiliation may also capture broader contextual factors, such as social integration and community cohesion.

In our study, we found a significant protective association between regional suicide rates and the incidence of Catholic populations. In addition, the risk of suicide was higher among non-religious individuals and those who did not respond. Both Catholic and Protestant religious traditions equally emphasise the prohibition of suicide. However, they differ in the degree of normative submission required from the individual to the Church hierarchy. Specifically, Protestant churches allow more room for individual interpretation. The classical Durkheimian framework emphasises two key mechanisms underlying suicide risk: social integration and moral regulation [16]. Catholicism, as a more hierarchical and institutionally cohesive religion, is characterised by both stronger social integration – through dense community networks and collective practices – and stronger moral regulation, reflected in clearly defined norms and authority structures.

In contrast, Protestant traditions, which emphasise individual interpretation of religious doctrine, are associated with lower levels of institutional integration and weaker normative regulation. According to Durkheim, this combination may increase vulnerability to social isolation and reduce the protective effects of collective moral frameworks.

In this context, the negative association observed between Catholic affiliation and suicide rates in our study may reflect these mechanisms of integration and regulation, rather than doctrinal differences per se.

Our findings suggest that non-religiosity, or individual religiosity not tied to historical churches representing traditional values, may not offer sufficient protection against certain psychological issues and life crises.

### Limitations

Census data on denominational affiliation are also the most reliable source available. The age-standardised incidence of denominational affiliation in the three censuses provides the most accurate characterisation of religiosity in Hungarian regions. Therefore, its use in quantile regression analyses best captures the relationship with age-standardised suicide rates at the population level. Additionally, quantile regression makes no assumptions about the distribution of variables and is resistant to the influence of outliers. Although the results of population-level ecological studies cannot be directly applied to individuals, the observed relationships warrant further investigation. Additionally, the use of lower (0.25) and upper (0.75) quantiles, along with Gamma regression in the sensitivity analyses, confirmed the main findings. In the quantile regression models (at the 50th percentile), the coefficients represent the change in the median age-standardised suicide mortality rate (ASSR) associated with a one-unit increase in the explanatory variable. In the Gamma models (with a log link), the coefficients reflect associations with the mean suicide mortality rate, providing a robust estimation that accounts for the heteroscedasticity and skewed distribution of the data.

In our study, we collected suicide data from the high-precision HCSO dissemination database since the turn of the millennium. Although data on religious denominations for suicides were unavailable, census data provide the best description of the population at risk. In addition to religious affiliations, several other factors may be related to suicide, but they were beyond the scope of this study due to the lack of structured data. Additionally, the regional prevalence of Catholicism may serve as a proxy for other unmeasured regional characteristics. However, Lantos and colleagues reported using the Hungarian dissemination database (HCSO’s Statinfo) that the risk of suicide was associated with gender, age group, region, marital status, and educational attainment, highlighting a nearly three-and-a-half times greater risk in males than in females overall [39].

A major limitation of our study is the substantial increase in non-response to the religious denomination question, rising from 10.8% in 2001 to 27.2% in 2011, and reaching 40.1% in 2022. This change is likely related to differences in census methodology, including the expansion of online self-completion and the voluntary nature of the question.

The increase in non-response may affect the comparability of religious affiliation across census years. If non-response is non-random—as suggested by its higher prevalence among younger and more urban populations—it may lead to underestimation of affiliation with historical churches [38, 40].

Although non-response increased across all regions, reducing the likelihood that regional differences are entirely artefactual, it may still bias the magnitude of observed associations. Therefore, the results should be interpreted with caution, particularly when assessing temporal trends in religious composition. Religious affiliation in this context may partly reflect response behaviour and broader processes of secularisation.

According to Freedman, Durkheim's idea that predominantly Protestant localities have higher suicide rates than predominantly Catholic localities is an example of the ecological fallacy [41]. The significant 15% decrease in Catholic affiliation and the ~ 30% increase in non-respondents may confound the results. Additionally, the potential for ecological fallacy is another limitation of our analysis: individual-level associations may not always reflect ecological-level associations. Some case–control studies have been conducted to analyse the relationship between suicide and religiosity; however, the choice of control group did not accurately characterise the risk population [42].

In our study, we examined the relationship between ASSR and ASIRs across different religious affiliations at a regional level. Only the regression coefficients were presented, as the risk estimation cannot be applied in this case due to the risk of ecological fallacy. Among our results, the negative relationship between regional ASSR and the standardised proportion of Catholics is noteworthy. Certainly, our results can only be used to generate hypotheses, which must be further tested using individual data. Although Zonda et al. analysed micro-regional data and reported a protective role of the Catholic religion in the risk of suicide [4], we can only conclude from the high-quality census data on religious denomination that regional ASSRs are higher where ASIRs for Catholics are lower, and ASIRs for non-religious individuals are higher.

## Conclusion

This study examined the relationship between suicide and religion. Our findings revealed that, using high-quality census data on religious denomination, regional ASSRs are higher where ASIRs for Catholics are lower, and ASIRs for non-religious individuals are higher.

Catholics, on the other hand, view salvation, faith, works, and sacraments as a process, placing authority in both the Bible and the traditions of the Catholic Church. They hold respect for the saints and the Pope, and the seven sacraments are central to Catholic belief and practice [43].

However, when considering religious or spiritual beliefs as protective factors against suicide, it is important to exercise caution. The protective value of religion and spirituality may stem from providing access to a socially cohesive and supportive community with a shared set of values [44]. However, this hypothesis requires further investigation.

## Data Availability

The data used in this study are available from: [https://statinfo.ksh.hu/Statinfo/themeSelector.jsp?&lang=en].

## Abbreviations

HCSO: Hungarian Central Statistical Office
ICD-10: International Classification of Diseases, 10th Revision
NUTS: Nomenclature of Territorial Units for Statistics
ASMR: Age-standardised mortality rate
ASIR: Age-standardised incidence rate
Catholics: Individuals affiliated with the Roman Catholic Church
Calvinists: Individuals affiliated with the Reformed Church of Hungary
Lutherans: Individuals affiliated with the Evangelical-Lutheran Church
HU11: Budapest region
HU12: Central Hungary/Pest County region
HU21: Central Transdanubia region
HU22: Western Transdanubia region
HU23: Central Transdanubia region
HU31: Northern Hungary region
HU32: Northern Great Plain region
HU32: Southern Great Plain region
RESP: Revised European Standard Population
CI: Confidence interval
SD: Standard deviation
